# Optimal Prediction of Moving Sound Source Direction in the Owl

**DOI:** 10.1371/journal.pcbi.1004360

**Published:** 2015-07-30

**Authors:** Weston Cox, Brian J. Fischer

**Affiliations:** 1 Department of Electrical and Computer Engineering, Seattle University, Seattle, Washington, United States of America; 2 Department of Mathematics, Seattle University, Seattle, Washington, United States of America; Duke University, UNITED STATES

## Abstract

Capturing nature’s statistical structure in behavioral responses is at the core of the ability to function adaptively in the environment. Bayesian statistical inference describes how sensory and prior information can be combined optimally to guide behavior. An outstanding open question of how neural coding supports Bayesian inference includes how sensory cues are optimally integrated over time. Here we address what neural response properties allow a neural system to perform Bayesian prediction, i.e., predicting where a source will be in the near future given sensory information and prior assumptions. The work here shows that the population vector decoder will perform Bayesian prediction when the receptive fields of the neurons encode the target dynamics with shifting receptive fields. We test the model using the system that underlies sound localization in barn owls. Neurons in the owl’s midbrain show shifting receptive fields for moving sources that are consistent with the predictions of the model. We predict that neural populations can be specialized to represent the statistics of dynamic stimuli to allow for a vector read-out of Bayes-optimal predictions.

## Author Summary

Many behaviors require predictive movements. Predictive movements are especially important in prey capture where a predator must predict the future location of moving prey. How sensory information is transformed to motor commands for predictive behaviors is an important open question. Bayesian statistical inference provides a framework to define optimal prediction and Bayesian models of the brain have received experimental support. However, it remains unclear how neural systems can perform optimal prediction in time. Here we use a theoretical approach to specify how a population of neurons should respond to a moving stimulus to allow for a Bayesian prediction to be decoded from the neural responses. This provides a novel theoretical framework that predicts properties of neural responses that are observed in auditory and visual systems of multiple species.

## Introduction

Predicting the future position of an object in the environment is a common and critical component of many tasks that involve reaching or orienting toward moving targets [1–4]. To execute these prediction tasks successfully, motor plans must extrapolate beyond accumulated sensory input to account for delays in sensory and motor processing, as well as for the future movements of the object. The ability to make accurate predictions of the location of a moving target is especially critical in prey capture.

Prey capture for moving targets has been studied at the behavioral and neural levels for animals that rely on visual [5–9] and auditory [10–12] information. For example, salamanders use visual input to predict the position of moving prey, make a head orienting movement toward the target, and then generate a ballistic movement of the tongue to capture the prey [7]. Barn owls also visually track their prey when possible [13], but are additionally able to use auditory information to capture moving prey [10]. After estimating a sound source’s trajectory, the owl makes a head orienting movement to localize a moving target before preparing to bring its feet forward to strike the prey [10]. Interestingly, salamanders and barn owls have neurons with similar specialized receptive fields that shift in time to mediate predictive prey capture [12,6,8]. These specializations occur in the fast-OFF retinal ganglion cells of the salamander [6,8] and the auditory spatially selective neurons in the optic tectum (OT) of the barn owl [12]. The receptive fields of these neurons shift toward a moving source, where the amount of shift is sufficient to account for delays in sensory and motor processing. Furthermore, it has been shown in the salamander that it is possible to read out the predicted location of a moving target from the fast-OFF retinal ganglion cells using a population vector average (PV) [8]. Here, we use the PV to model the computations performed by the barn owl as it tracks a moving sound source and address how such a neural circuit may approach optimal performance.

These studies open several questions about the neural basis of predictive behaviors. What information is represented in these populations of neurons? Is the observed neural representation an optimal solution to the prey capture problem faced by each species? An optimal solution to the prediction problem would take into account the source dynamics, sensory statistics, and prior information to guide the solution. This approach to an optimal solution can be formulated as Bayesian prediction [14]. There is support for Bayesian models of perception and behavior in diverse tasks across multiple species [15–18]. Additionally, there have been multiple proposals for how neural systems can implement Bayesian inference [19–23,16,24–26]. In particular, several studies have addressed the problem of inference in time in the context of hidden Markov models [20,27,28] and tracking using the Kalman filter [29,19,22,30,31]. However, it remains unknown how a neural system can perform Bayesian prediction.

Here we specify how a population of neurons should respond to a moving stimulus to allow for a Bayesian prediction to be decoded from the neural responses. We approach this question in the context of auditory-based prey capture by the barn owl. The Bayesian prediction problem we consider is that of predicting a sound source’s future direction, given a sequence of sensory observations and a prior distribution for direction and angular velocity. It has been shown that the owl’s sound localization for brief sounds is consistent with a Bayesian model [24]. Here, evolutionary pressure for optimality may be expected, given the dependence of owls on successful sound localization during hunting.

The success of the PV in decoding predictive movements of visual targets in the salamander [8] and dragonfly [32] makes this a viable candidate mechanism for implementing Bayesian prediction. It has been shown that the owl’s map of auditory space decoded by a PV is consistent with the owl’s localization behavior for brief stationary stimuli [24]. More generally, it has been shown that a population code can encode the statistical properties of the environment to allow a PV to match a Bayesian estimator [23,26,24]. This model for the neural implementation of Bayesian inference is attractive because it matches the common observation that population codes are adapted to natural statistics [33]. However, the applicability of the PV model to Bayesian inference in time is unknown. Here, we determine the conditions under which a population of neurons with spatial-temporal receptive fields can perform Bayesian prediction for moving sound sources.

## Results

### Bayesian prediction

**Fig 1:**
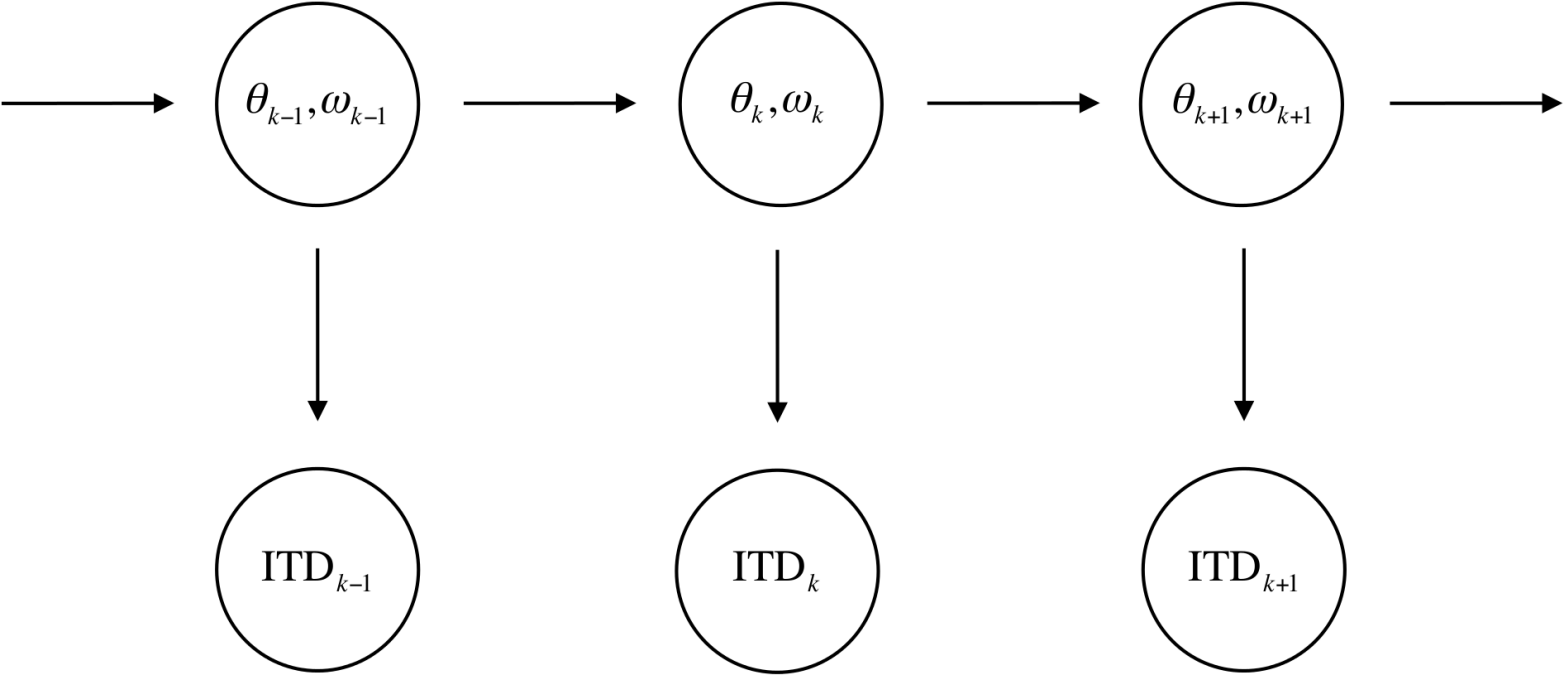
Graphical model describing the prey capture problem. The unobserved state of the prey is described by the direction *θ*_*k*_ and the angular velocity *ω*_*k*_ at time *k*. The state at time *k* is conditionally independent of states at previous times *i* given the value of an intermediate state at time *j*, where *i* < *j* < *k*. The observed sensory input at each time is the interaural time difference (ITD), which only depends on the state of the source at that time step.

We consider the problem of predicting the future direction of a moving source from a temporal sequence of auditory observations. Specifically, the prey capture problem is that of predicting the direction of a moving sound source a short time in the future based on the sequence of interaural time difference (ITD) measurements from the sounds reaching the left and right ears (Fig 1). ITD is the difference in the arrival time of sounds at the two ears and is a primary cue for localization in the horizontal dimension [34,35].

The Bayesian filtering approach to predicting at time *k* the direction at a point *n* time steps in the future *θ*_*k*+*n*_, given the sequence of observations up to time *k*, *ITD*_1:*k*_ = [*ITD*_1_, *ITD*_2_, … *ITD*_*k*_], is to compute an estimate from the posterior distribution *p*_*k*+*n*_(*θ*, *ω*|*ITD*_1:*k*_). The form of the posterior distribution is determined by a model for the dynamics of the moving target and the statistical relationship between the state of the target and the ITD observations.

### Generative model

The temporal dynamics of the horizontal direction of the moving target are modeled as

$$\theta_{k}=\theta_{k-1}+\Delta t\omega_{k-1}+\eta_{k}$$

$$\omega_{k}=\omega_{k-1}+\nu_{k}$$

where *θ*_*k*_ is the target direction, *ω*_*k*_ is the angular velocity, *η*_*k*_ is a zero-mean circular Gaussian noise process, *ν*_*k*_ is a zero-mean Gaussian noise process, *k* is the current time step, and Δ*t* is the time step duration. The sensory information ITD is modeled as a sinusoidal function of direction that is corrupted by noise:

$${ITD}_{k}=A\;\text{sin}\left(2\pi f\theta_{k}\right)+\;\xi_{k}$$

where *ξ*_*k*_ is a zero-mean Gaussian noise process with standard deviation 12.5 μs and the amplitude *A* and frequency *f* are determined by the shape of the owl’s head and facial ruff [24]. The sinusoidal mapping between direction and ITD is based on direct measurements of ITD for the barn owl [36]. All noise processes are assumed to be mutually independent and uncorrelated across time.

The noise process *ν*_*k*_ influencing the prey velocity depends on the type of behavior displayed by the prey. A large standard deviation of the noise corresponds to irregular fleeing behavior displayed by prey under close attack when there is no place to hide [37]. A small standard deviation produces a smoother trajectory for the prey, which corresponds to escape toward cover [37]. Here we use a velocity-noise standard deviation of 0.125 deg/s corresponding to mouse escape behavior under close-distance owl attack where prey trajectories are smooth [37]. This parameter value has the effect of keeping the velocity roughly constant over a short period of time.

The prior depends on both the natural prey behavior and the owl’s bias as determined by the behavioral cost function [24]. Here we assume that the prior emphasizes directions at the center of gaze [24] and slow source velocities. We also assume that there is a weak negative correlation between direction and velocity such that there is a bias for sources moving into the center of gaze [38,39]. The form of the prior is a Gaussian with zero mean for both direction and velocity. The standard deviation for direction is 23.3 deg [24], the standard deviation for velocity is 50 deg/s, and the correlation between direction and velocity is -0.05. The parameter values for the velocity standard deviation in the prior ($\sigma_{v_{0}}=50$ deg/s) and during movement ($\sigma_{v_{k}}=0.125$ deg/s, *k* ≥ 1) describe a situation where the initial velocity can take on a wide range of values, but the velocity will be roughly constant over a short period of time.

### Bayesian tracking and prediction

**Fig 2:**
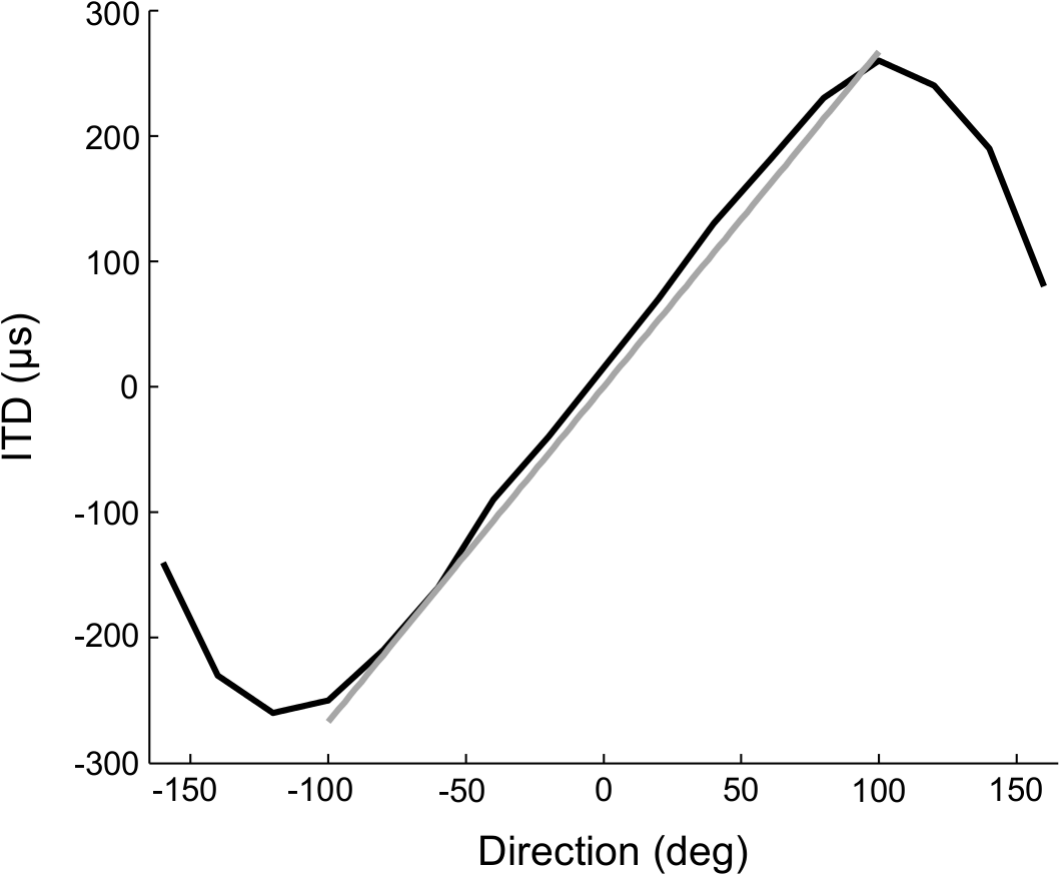
Linear approximation to ITD. The measured relationship between direction and ITD is approximately sinusoidal (black curve; [39]). The linear approximation *ITD* = 2.67 *μs* / deg × *θ* (gray line) accurately describes the relationship between direction and ITD over the frontal hemisphere.

The Bayesian prediction at time *k* of the direction at a point *n* steps in the future, *θ*_*k*+*n*_ given the sequence of observations *ITD*_1:*k*_ is computed as the mean of the posterior *n* steps in the future *p*_*k*+*n*_(*θ*, *ω*|*ITD*_1:*k*_) Because we are estimating a circular variable, the Bayesian prediction is the direction of the Bayesian prediction vector, defined as the vector that points in the direction of the mean value of the direction *n* steps in the future:

$${BV}_{k}=\;\int \;u\left(\theta \right)p_{k+n}\left(\theta |{ITD}_{1:k}\right)d\theta ,$$

where *u*(*θ*) is a unit vector pointing in direction *θ* (Methods). Solving the Bayesian prediction equations may be computationally difficult for nonlinear or non-Gaussian models [40]. If the system is linear with Gaussian noise, then the Kalman filter can be used for Bayesian prediction [41]. Our model includes Gaussian noise but the mapping from direction to ITD is nonlinear. We found that relationship between direction and ITD is nearly linear for sound sources in the frontal hemisphere (Fig 2). The root-mean-square (RMS) error between the measured ITD and the linear approximation *ITD* = 2.67 *μs* / deg × *θ* was 15.1 μs for directions between -100 deg and 100 deg. We therefore used the Kalman filter to perform Bayesian prediction for computational simplicity (Methods).

**Fig 3:**
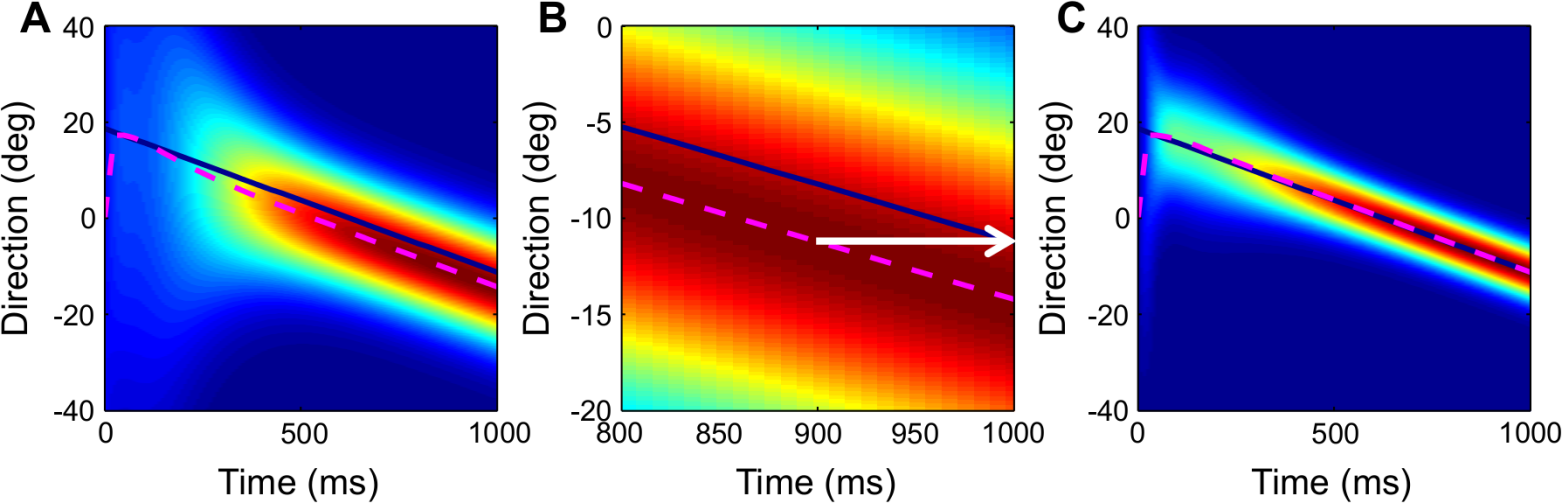
Bayesian prediction and tracking. (A) The predictive posterior is shown for a source trajectory starting at 20 deg to the right and moving across the frontal hemisphere. Red indicates regions of high probability density and blue indicates regions of low probability density. The center of the predictive posterior (pink dashed line) initially trails the stimulus, but then leads the source by approximately 100 ms. (B) The predictive posterior zoomed in near the peak between 800 and 1000 ms from (A) shows the prediction leading the source by approximately 100 ms. (C) The posterior for tracking is similar to the posterior for prediction, but is used to estimate the current, not future, direction of the source.

The Bayesian model successfully predicts future directions of the prey for smoothly moving sources (Fig 3). We chose the prediction time step *n* in order to predict the source direction 100 ms in the future [12]. Initially the Bayesian prediction is dominated by the prior distribution, which emphasizes central directions (Fig 3A). Because of the influence of the prior, the posterior does not initially lead the source direction. However, after a short delay the posterior *p*_*k*+*n*_(*θ*, *ω*|*ITD*_1:*k*_) predicts the future direction of the source (Fig 3A and 3B). Note that the performance of the Bayesian prediction differs from the Bayesian tracking estimate. Whereas the tracking algorithm seeks to place the center of posterior at the current source position (Fig 3C), the prediction algorithm seeks to place the center of posterior at the future position of the source. Also, the predictive posterior (Fig 3A) is wider than the posterior for tracking (Fig 3C) because uncertainty increases as the time window for prediction increases beyond the current time where observations are available.

### Population vector implementation

It has been shown that the owl’s map of auditory space decoded by a PV is consistent with the owl’s localization behavior for brief stationary sounds [24]. Here we investigate conditions on a population of neurons with spatial-temporal receptive fields under which the PV will match the Bayesian prediction in time. The PV at time *k* is given by an average of weighted preferred direction vectors:

$${PV}_{k}=\frac{1}{N}\sum_{j=1}^{N}a\left({ITD}_{1:k}|\theta^{\left(j\right)},\omega^{\left(j\right)}\right)u(\theta^{\left(j\right)})$$

where the preferred directions *θ* ^(*j*)^ are defined by the motor output. The PV at time *k* depends on the sequence of past ITD measurements and predicts the future direction of the target. By associating each neuron with a fixed preferred direction *θ* ^(*j*)^, we are making the assumption that the motor neurons that the OT neurons ultimately influence are fixed. This assumption means that the effect of a given level of response for an OT neuron on the motor output stays constant. The rate function *a*(*ITD*_1:*k*_|*θ* ^(*j*)^, *ω* ^(*j*)^) is the firing rate of the *j* ^*th*^ neuron in response to the sequence of ITD values *ITD*_1:*k*_. We now state our main result, which specifies sufficient conditions so that the PV will approximate the Bayesian prediction estimate.

#### Proposition

If the neural activities satisfy

$$a\left({ITD}_{1:k}|\theta^{\left(j\right)},\omega^{\left(j\right)}\right)=\;\alpha \frac{p_{k+n}\left(\theta^{\left(j\right)},\omega^{\left(j\right)}|{ITD}_{1:k}\right)}{q(\theta^{\left(j\right)},\omega^{\left(j\right)})}$$

for neurons with indices *j* = 1, 2, … *N*, where α does not depend on *θ* or *ω*, and the preferred directions *θ* ^(*j*)^ and angular velocities *ω* ^(*j*)^ are drawn from the proposal density *q*(*θ* ^(*j*)^, *ω* ^(*j*)^), then the PV converges to a vector pointing in the same direction as the Bayesian prediction vector as the number of neurons *N* approaches infinity.

Proof: Suppose that the neural activities satisfy

$$a\left({ITD}_{1:k}|\theta^{\left(j\right)},\omega^{\left(j\right)}\right)=\;\alpha \frac{p_{k+n}\left(\theta^{\left(j\right)},\omega^{\left(j\right)}|{ITD}_{1:k}\right)}{q(\theta^{\left(j\right)},\omega^{\left(j\right)})}$$

for *j* = 1, 2, … *N* and that the preferred directions *θ* ^(*j*)^ and angular velocities *ω* ^(*j*)^ are drawn from the density *q*(*θ* ^(*j*)^, *ω* ^(*j*)^). As the number of neurons in the population *N* approaches infinity, the PV converges to an expected value

$${PV}_{k}\to \iint \;u\left(\theta \right)q\left(\theta ,\omega \right)a\left({ITD}_{1:k}|\theta ,\omega \right)d\omega d\theta .$$

With the neural activities defined as the ratio of the posterior to the proposal density, the PV converges to

$${PV}_{k}\to \iint \;u(\theta )q(\theta ,\omega )a({ITD}_{1:k}|\theta ,\omega )d\omega d\theta \propto \iint \;u\left(\theta \right)p_{k+n}(\theta ,\omega |{ITD}_{1:k})d\omega d\theta =\;{BV}_{k}$$

and thus points in the same direction as the Bayesian prediction vector.

A special case of this result occurs when the proposal density from which the preferred stimuli are drawn is the prior density. The more general result stated in the proposition allows for the distribution of preferred stimuli to possibly have more neurons in the periphery than would be predicted by the prior as long as the neural responses compensate for this increase in the number of neurons by decreasing the gain of the responses in the periphery. Current experimental evidence, however, is consistent with the distribution of preferred directions matching the prior distribution [24]. In particular, for a brief static stimulus, the special case corresponds to the preferred stimuli being drawn from the prior and the neural responses being proportional to the likelihood function, for which there is experimental support [24,42]. We therefore predict that neural responses supporting Bayesian prediction in time are given by a ratio of the posterior at a time *n* steps in the future to the prior distribution. In the following we explore the implications of this prediction for receptive field properties of neurons supporting Bayesian prediction.

### Receptive field shifts

**Fig 4:**
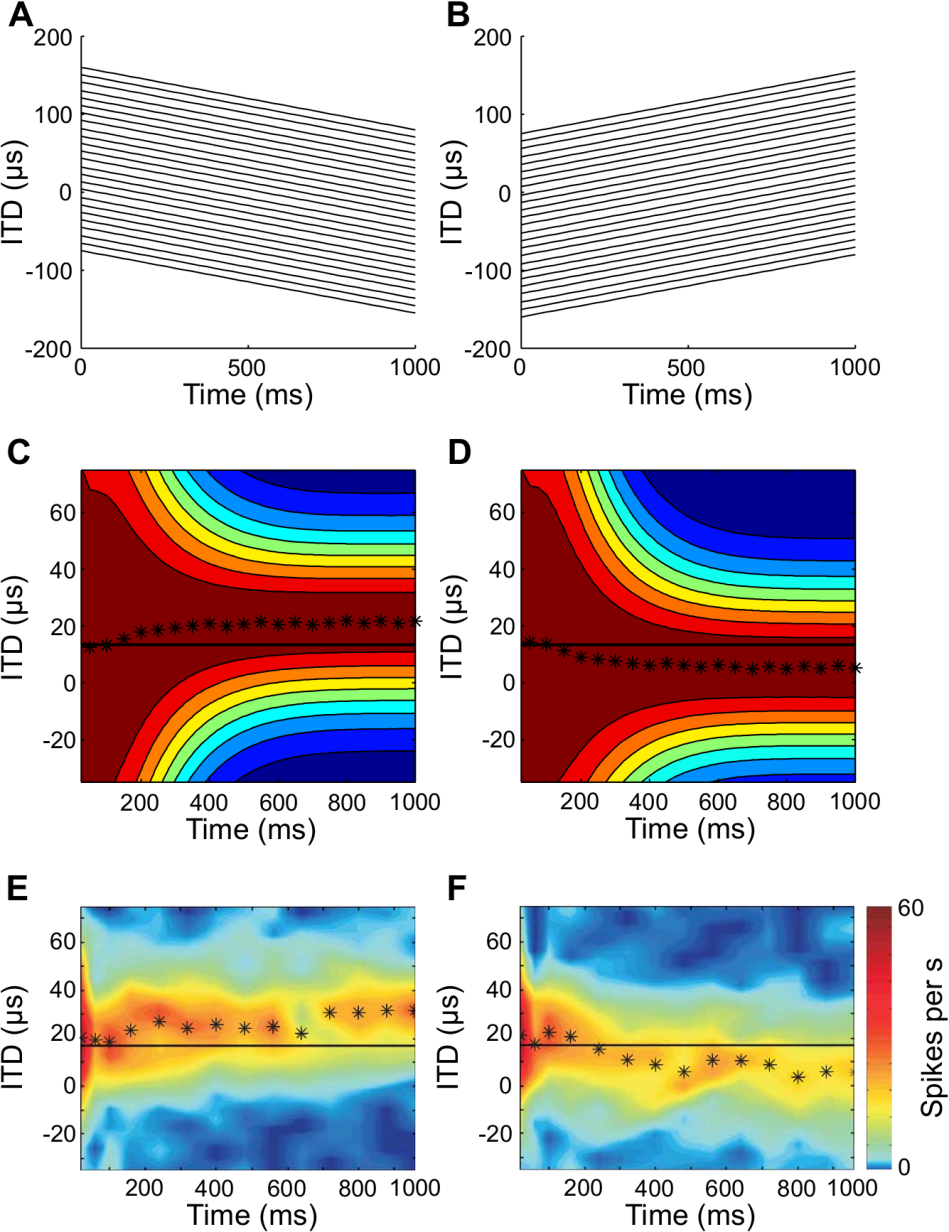
Predicted and measured receptive field shifts. (A,B) Stimulus trajectories moved at a constant velocity across the frontal hemisphere. (C,D) Predicted receptive field shifts from the model for rightward (C) and leftward (D) motion. The solid line represents the preferred ITD for static stimuli. The black stars represent the peak ITD of the response at the given time. Color codes for firing rate, with red indicating a high firing rate and blue indicating a low firing rate. (E,F) Experimentally measured receptive field shifts of an optic tectum neuron from [12]. Reprinted by permission from Macmillan Publishers Ltd: Nature Neuroscience [12].

**Fig 5:**
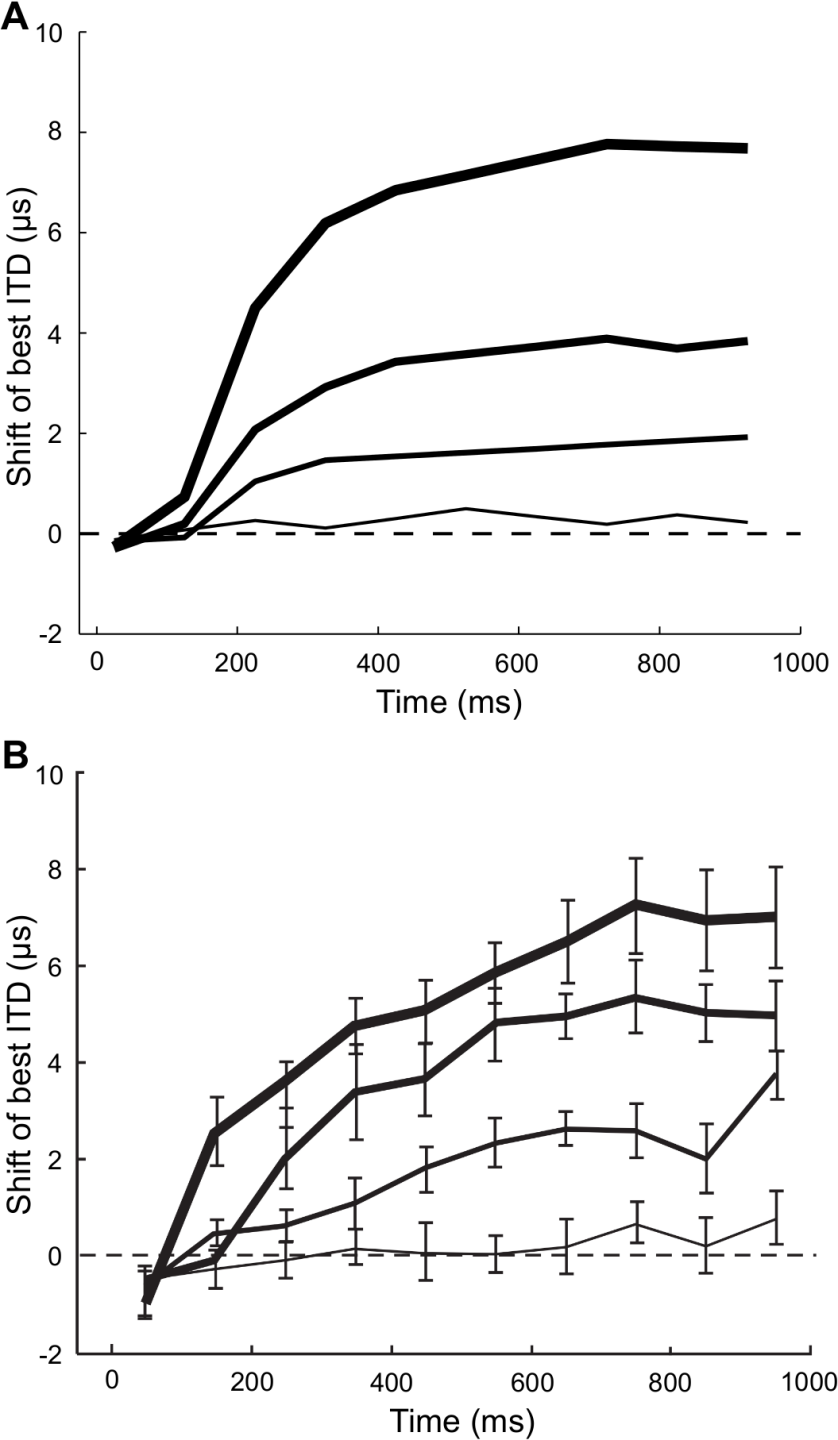
Receptive field shifts and velocity. (A) Shifts of the best ITD as a function of time for different source velocities. Faster velocities are coded by thicker lines (5 μs ITD/s, 20 μs ITD/s, 40 μs ITD/s, 80 μs ITD/s). (B) Shifts of best ITD as a function of time for different source velocities of optic tectum neurons from [12]. Reprinted by permission from Macmillan Publishers Ltd: Nature Neuroscience [12].

The first prediction derived from our result is that neurons implementing Bayesian prediction using this type of population code will have receptive fields that shift in time towards the moving source (Fig 4A–4D). This is the type of shift that is necessary to compensate for delays and allow for the owl to capture the moving source [6,12,8]. These delays include signal processing in the brain as well as motor delays, and total approximately 100 ms [12]. While the receptive fields shift in time, there is a delay to the onset of the shift of the receptive field. This delay in the shift occurs in the Bayesian model because the response is initially dominated by the prior before sufficient sensory information has been accumulated. Therefore, the predictive posterior initially lags behind the source direction (Fig 3A). It is only after a delay that the predictive posterior leads the current source direction. The model also predicts that receptive fields get sharper with time. The sharpening of the receptive fields follows the sharpening of the posterior as more sensory information is collected (Fig 3A). Additionally, the model predicts that the shift of the receptive field depends on the speed of the moving source. Faster source velocities lead to larger shifts, while slower source velocities correspond to smaller shifts of the receptive field (Fig 5A). This prediction follows from the fact that the posterior shifts faster for faster sources.

The receptive field shifts predicted by the model are consistent with experimental results in the barn owl [12] (Figs 4 and 5). Neurons in the owl’s OT that are involved in generating head orienting movements show shifting receptive fields for moving sources [12] (Fig 4E and 4F). The receptive field shifts in the owl are consistent with the Bayesian prediction model in that the shift toward the source is not instantaneous, but occurs after a delay (Fig 4E and 4F). Receptive fields of midbrain neurons also get sharper in time, as predicted by the model [12,43]. Additionally, the size of the shift varies with the speed of the moving source (Fig 5B). The time course and magnitude of the observed shifts correspond well to the predicted shifts in the model.

**Fig 6:**
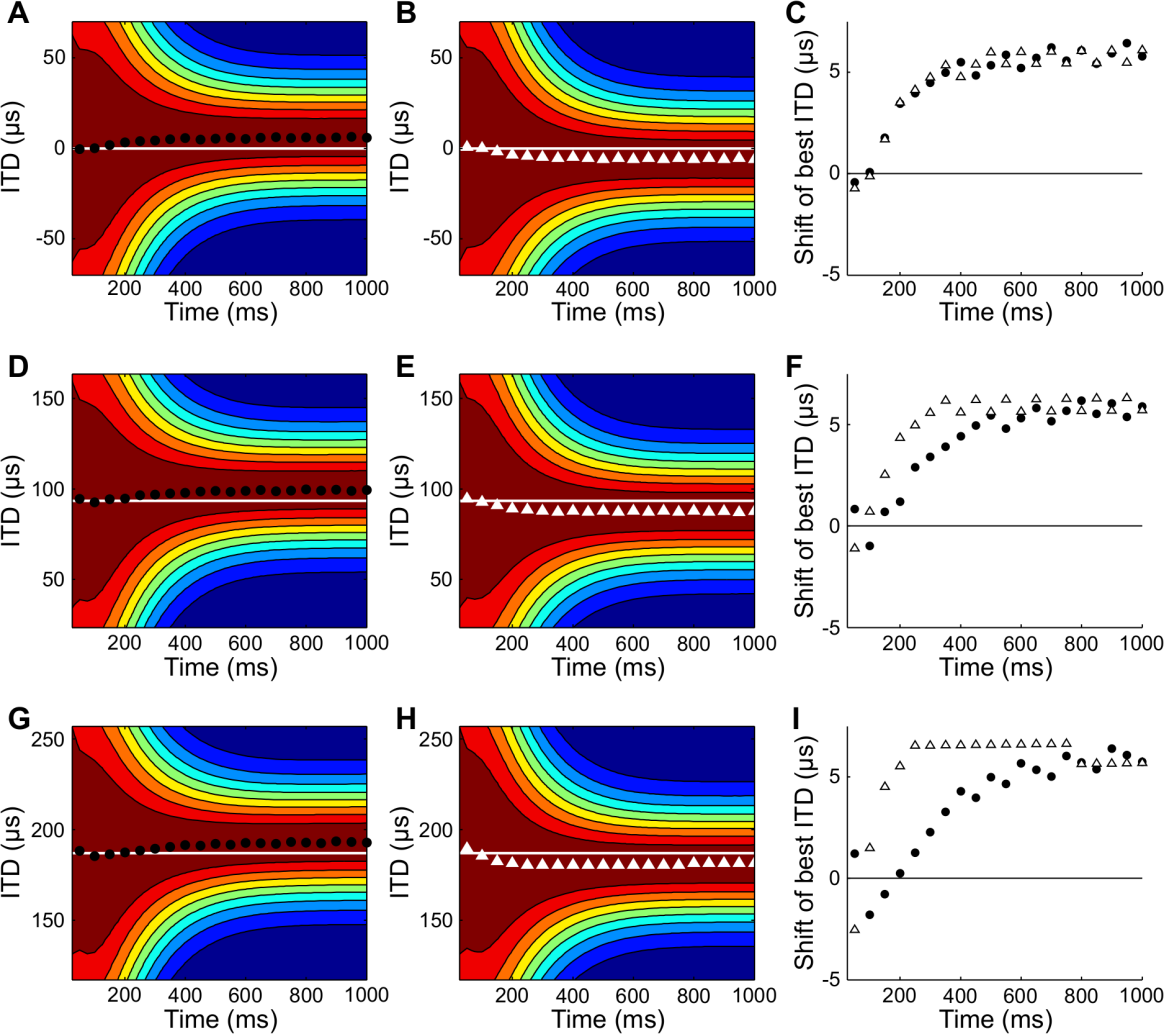
Asymmetry in predicted receptive field shifts. (A,B) Receptive field shifts for counterclockwise (A) and clockwise (B) moving sources for a neuron with preferred direction 0 deg. (C) The shift of best ITD is symmetric for the two directions. The shift is plotted as measured for the counterclockwise motion and is the negative of the shift for clockwise motion, for comparison between the cases. (D-F) The shifts are asymmetric for a neuron with preferred direction 35 deg. (G-I) The asymmetry is more pronounced for a neuron with preferred direction 70 deg.

The model predicts an asymmetry in the shifts of the receptive fields for sounds moving into and out of the center of gaze that increases with the eccentricity of the receptive field (Fig 6). For neurons with receptive fields at the center of gaze, the shifts for clockwise and counterclockwise sources are mirror images (Fig 6A–6C). For neurons with more peripheral receptive fields, the shifts for clockwise and counterclockwise moving sources are asymmetric (Fig 6D–6I). For neurons with peripheral receptive fields, the initial shift of the receptive field for sources moving into the center of gaze is in the opposite direction than one would expect based on the idea that receptive fields should move towards the source. This occurs because of the effect of the prior on the performance of the posterior (Fig 3A). Initially, the posterior is dominated by the prior and thus at stimulus onset is not leading the source by the desired 100 ms.

The asymmetry of the receptive field shifts for peripheral OT neurons has not been investigated in the owl. However, neurons in the owl’s external nucleus of the inferior colliculus (ICx) do have an asymmetry in their direction selectivity for sounds moving into and out of the center of gaze, which may be related to asymmetric shifts [38,39]. Testing this prediction will require further study.

**Fig 7:**
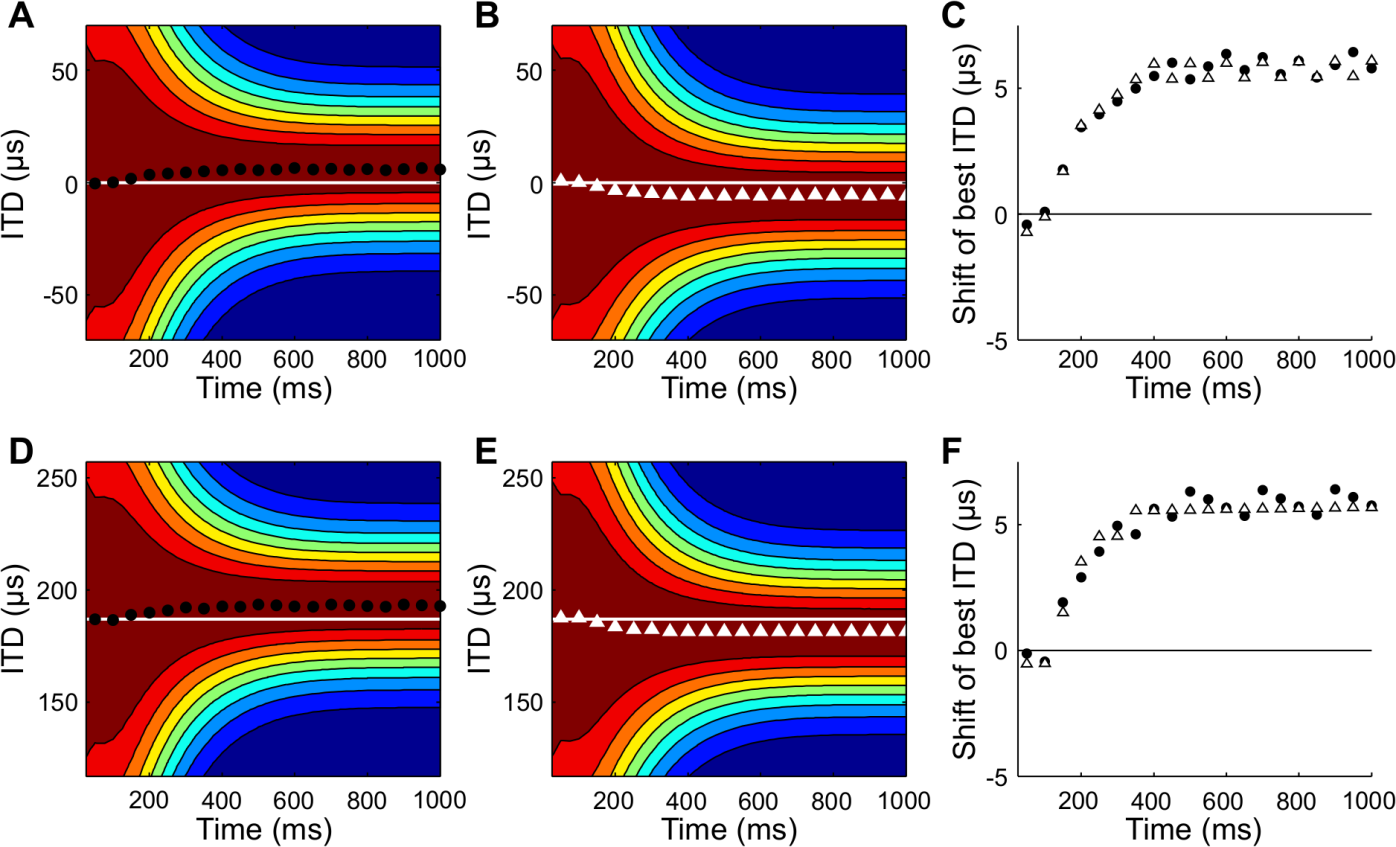
Asymmetry in predicted receptive field shifts with a uniform prior distribution. Receptive field shifts for counterclockwise and clockwise moving sources, as in Fig 6, for neurons with preferred direction 0 deg (A-C) and 70 deg (D-F) when the prior is a uniform distribution.

The prediction of asymmetry in the receptive field shift for clockwise moving and counterclockwise moving sources depends on the presence of a prior that emphasizes central directions. We found that predicted receptive field shifts were symmetric for clockwise moving and counterclockwise moving sources in both central and peripheral neurons when the prior in the model was uniform (Fig 7). As noted above, the asymmetry is caused by the initial dominance of the prior on the location of the peak in the posterior. When the prior is uniform, this effect is removed and the posterior can quickly lead the source direction for motions both into and out of the center of gaze.

**Fig 8:**
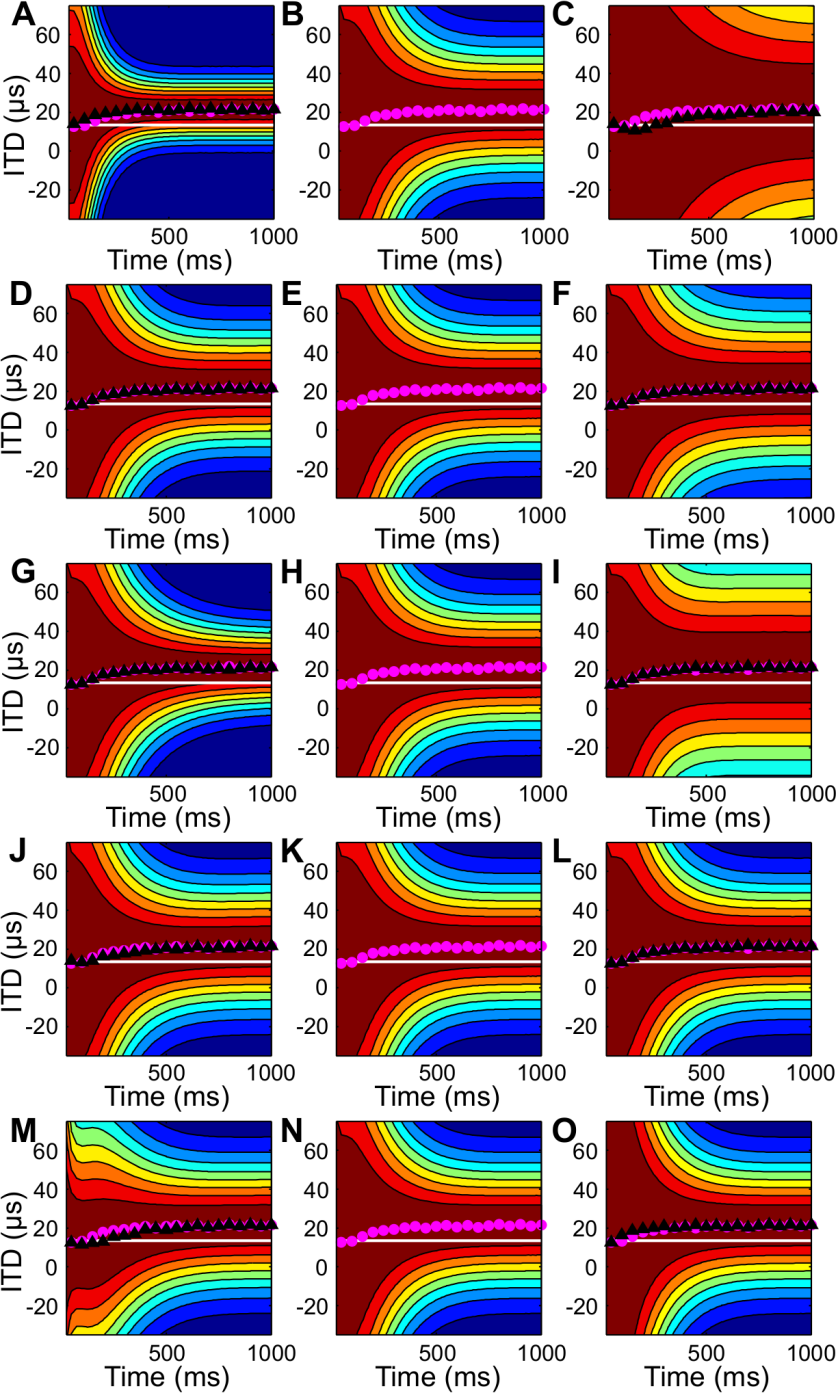
Parameter dependence of the receptive field shifts. Receptive field shifts are shown for a neuron with preferred direction 0 deg for parameter values at the selected values (center column), half this value (left column) and twice the value (right column). The pink dots show the best ITD at the parameter values used in other figures and are the same for all panels. Parameters were changed individually and included (A-C) the standard deviation of the ITD noise, (D-F) the standard deviation of the direction noise in the dynamics model, (G-I) the standard deviation of the velocity noise in the dynamics model, (J-L) the standard deviation of the direction in the prior, and (M-O) the standard deviation of the velocity in the prior.

The receptive field shifts predicted by the model were robust to parameter variation (Fig 8). We examined the receptive field shifts for different standard deviations of the noise terms and different prior standard deviations for direction and velocity. The model predicted similar magnitudes of shifts for the chosen values (center column) and when each parameter was halved (left column) or doubled (right column). Changing the standard deviation of the noise corrupting ITD had the greatest effect on the receptive fields (Fig 8A–8C). This parameter influences the width of the posterior and therefore influences the width of the receptive field.

### Population decoding

**Fig 9:**
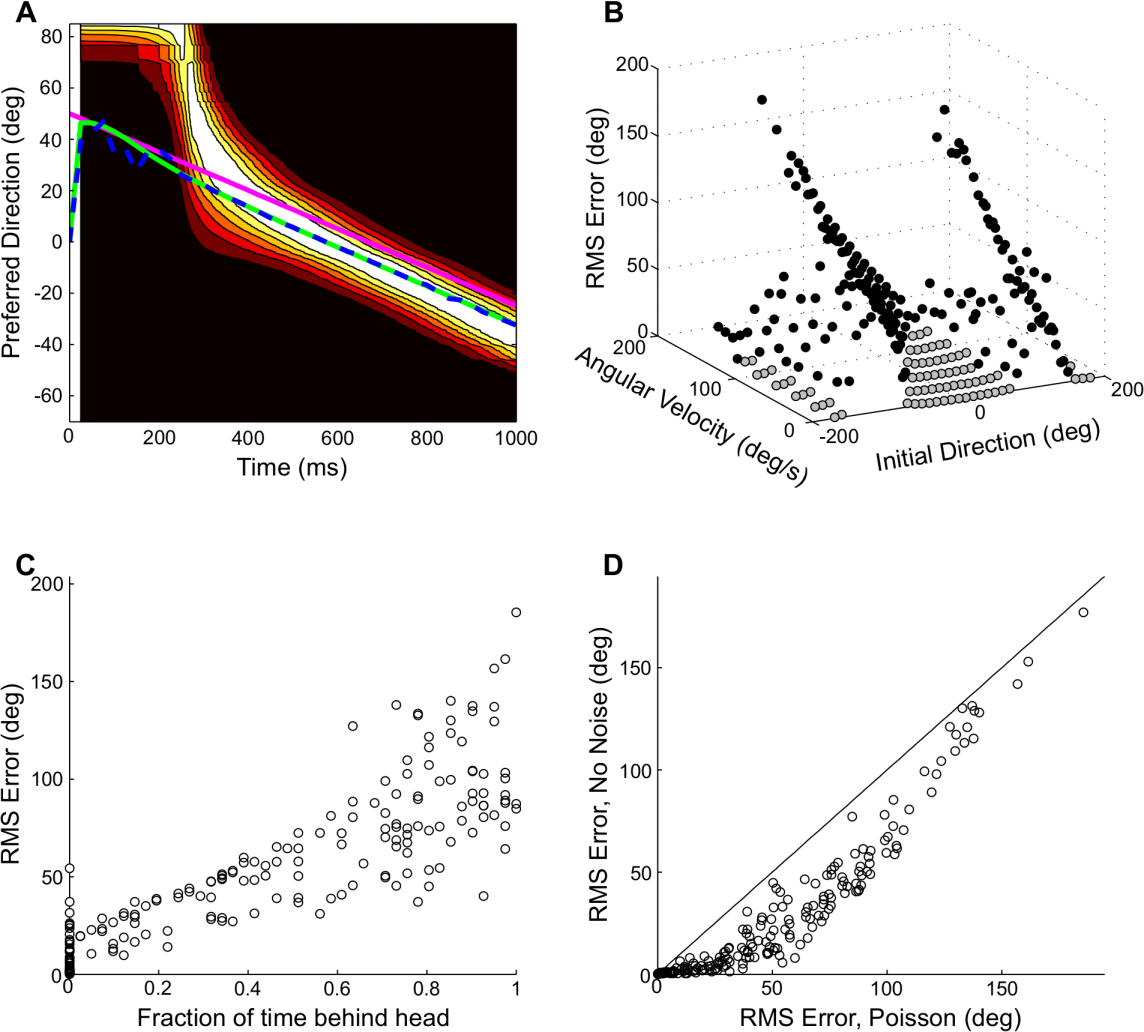
Population decoding of predicted source direction. (A) Example model population activities plotted together with the true source direction (pink), the Bayesian prediction direction (green) and the PV estimate (blue). The color map codes for firing rate with white and black indicating high and low firing rates, respectively. (B) RMS error between the Bayesian prediction and the PV estimate averaged over one-second duration stimuli at the given initial direction and angular velocity. The values in gray indicate stimulus conditions where the RMS error was less than 3 deg. (C) The RMS error is highly correlated with the fraction of time the predicted source direction is behind the head. This occurs because all preferred directions are in the frontal hemisphere. (D) RMS error between the Bayesian prediction and the PV estimate for deterministic neurons and Poisson neurons. The solid line is the identity line.

The net effect of the receptive field shifts is that the activity moves across the population so that it predicts the future direction of the moving source (Fig 9A). It is this activity that must be decoded by the PV to approximate the Bayesian prediction. To test the PV implementation of Bayesian prediction, we constructed a model of 5000 Poisson neurons with receptive fields that shift according to the posterior (Methods). The PV matched the Bayesian prediction closely for different stimulus conditions (Fig 9). The PV approximated the Bayesian prediction to within 3 degrees (root-mean-square (RMS) error) for velocities up to 125 deg/s (Fig 9B). The RMS error in the approximation of the Bayesian prediction by the PV depended strongly on the fraction of time the predicted source direction was in the frontal hemisphere (spearman rank correlation = 0.92; Fig 9C). Since all of the preferred directions of the model neurons are in the frontal hemisphere, the model will necessarily fail when the posterior is localized at source directions behind the head. We also computed the RMS error using a population of deterministic neurons to determine the contribution of the Poisson variability of the neurons to the error (Fig 9D). The Poisson variability increased the RMS error for many trajectories (mean ± s.d. ratio of RMS error for deterministic neurons to RMS error for Poisson neurons 0.43 ± 0.23). However, the largest errors in the approximation are primarily due to the limited range of preferred directions of neurons in the population. The pattern of error as the initial direction and velocity of a moving source varied is explained by larger errors occurring when the predicted source trajectory spends more time behind the head.

## Discussion

We showed that the PV can read out the Bayesian prediction in time from a population of neurons. The PV will approximate the Bayesian prediction when the population has specialized responses with shifting receptive fields. The types of shifting receptive fields predicted by our analysis are observed in the OT of the owl [12] and the retina of the rabbit [6] and salamander [6,8]. This result shows that with the appropriate encoding of the stimulus, a simple decoding algorithm can perform complex computations [44,19,8].

Our work provides a theoretical framework in which to interpret observations about circuits underlying prediction. Previous work identified neurons in the OT [12] and retina [6,8] with shifting receptive fields that account for delays in neural processing. Leonardo and Meister (2013) further showed that decoding a population of such responses with a PV can predict a moving target position. Our work shows that this type of network computation can be optimal and capture the statistics of a dynamic target.

This work shows that a non-uniform population code model with a PV decoder can implement Bayesian inference for stationary and moving sources. The non-uniform population code model proposes that a prior distribution is encoded in the distribution of preferred stimuli and that the statistics of the sensory input are encoded by the pattern of neural responses across the population [23,24]. Here we extend this model to show that the dynamics of a population code can represent the statistics of a dynamical system. This is an important extension of the non-uniform population code model due to the dynamic nature of ethologically relevant stimuli.

We make several predictions about the receptive field shifts necessary for optimal prediction. First, we predict that neurons have receptive fields that shift towards a moving source where the shift increases with the source velocity. This prediction is consistent with observations in the OT [12] and retina [6,8]. We also predict that the shift is sluggish when a non-uniform prior is present. This is consistent with responses of OT neurons [12]. Our analysis also leads to several predictions that have not been tested in the auditory or visual systems. In particular, we predict an asymmetry in the shifts of receptive fields for sources moving into and out of the center of gaze when a prior emphasizes the center of gaze (Fig 6). We also predict that for noisier stimuli, the magnitude of the shift will decrease and the receptive fields will become wider (Fig 8A–8C). Finally, we predict that receptive fields should become narrower over time to reflect the accumulation of sensory information. Studies of neurons thought to support predictive behaviors have not yet investigated all of these response features predicted by our model.

Bayesian theories of perception propose that neural systems represent statistical models of the environment, where the models may contain many parameters. The parameters of these models may be learned by an animal over multiple time scales. For the owl, information about the prior and the basic relationship between sound localization cues and source directions is primarily due to a combination of genetic changes over an evolutionary time scale and learning over the life of the animal [45]. There is evidence, however, that the owl adjusts to the noise level of the stimulus on a trial-to-trial basis [46]. We therefore predict that the noise-level parameter of the model is learned rapidly, leading to wider and more slowly shifting receptive fields in high noise environments. Future work is required to determine how the parameters of the model are learned in the owl’s auditory system.

Previous studies have shown that a cascade model with a gain control component can produce the experimentally observed shifting receptive fields [6,12,8]. This model involves a negative feedback loop, causing the neural response at each time step to be influenced by its predecessors. This model is phenomenological, but it suggests that a recurrent network within the OT is sufficient to generate the receptive field shifts necessary for Bayesian prediction. However, neurons upstream from OT in ICx show direction selectivity [39,47] and it is therefore possible that shifting receptive fields originate in ICx. Furthermore, the asymmetric direction selectivity observed in ICx may possibly be explained by single-cell adaptation [39] rather than by a network effect. Therefore, the mechanism underlying receptive field shifts in OT remains an open question.

Previous work has addressed inference in time using the Kalman filter [19,22,30,48]. While we determine how a population of neurons should respond to a moving stimulus but did not specify a mechanism for implementing the responses, these studies constructed networks to represent the Kalman filter estimate and variance as a function of time. One type of model produces a population code where the estimate of the target location is at the peak of a symmetric population response [22,30]. This is accomplished through a nonlinear encoding model involving divisive normalization. It is possible to read out the estimate using a center-of-mass decoder, but the model is limited to Gaussian distributions. Another model encodes the target estimate and variance using a linear probabilistic population code [48]. This model also relies on divisive normalization to implement the Kalman filter, but requires a nonlinear decoder to determine the estimated location from the activities. The model of Eliasmith and Anderson (2003) utilizes nonlinear responses and linear decoding. However, unlike the preferred direction vectors in the PV, the linear decoders are not in general equal to the preferred directions and are obtained using supervised learning. These models may be extended to consider the case of prediction, but the responses of neurons performing prediction in these schemes has not been investigated. Our model differs from the previous models in that the preferred direction at the peak of the population activity profile will not in general equal the PV estimate (Fig 9). This occurs because our model includes a non-uniform population, whereas previous models use a uniform population. An additional distinction between our model and previous models is that our predictions apply to nonlinear and non-Gaussian models.

It has previously been shown that the PV performs poorly when decoding arm movements from motor cortical responses [49]. The work presented here does not conflict with this previous finding. We show that the PV will perform well in tracking and prediction when the receptive fields of the neurons encode the state dynamics with shifting receptive fields. This is not a general-purpose decoder, but rather must be used to read out the activity of a specialized population with shifting receptive fields such as those in the OT. Experimental evidence suggests that populations of neurons with response properties that are adapted to the natural statistics are important for perception and behavior. The work presented here shows how network properties tailored to the dynamics of moving prey allow for optimal Bayesian prediction by a population of neurons.

## Methods

### Bayesian prediction equations

The Bayesian prediction at time *k* of the direction at a point *n* steps in the future *θ*_*k*+*n*_, given the sequence of observations *ITD*_1:*k*_ is computed from the posterior *n* steps in the future *p*_*k*+*n*_(*θ*, *ω*|*ITD*_1:*k*_). To construct the posterior at time *k*+*n* we first compute the posterior at the current time step *p*_*k*_(*θ*, *ω*|*ITD*_1:*k*_), then predict *n* steps in the future using the transition probability density *p*_*k*+*n*|*k*_(*θ*_*k*+*n*_, *ω*_*k*+*n*_|*θ*_*k*_, *ω*_*k*_). Using the dependence relationships between direction, velocity, and ITD indicated in Fig 1, the posterior at time *k*+*n* is given by

$$p_{k+n}\left(\theta ,\omega |{ITD}_{1:k}\right)=\;\iint \;p_{k+n|k}\left(\theta ,\omega |\theta_{k},\omega_{k}\right)p_{k}\left(\theta_{k},\omega_{k}|{ITD}_{1:k}\right)d\theta_{k}d\omega_{k}.$$

The Bayesian prediction of the direction of the sound source at time *k*+*n* conditioned on the observations *ITD*_1:*k*_ is the mean of the predictive posterior over direction *p*_*k*+*n*_(*θ*|*ITD*_1:*k*_). This posterior is found by marginalizing *p*_*k*+*n*_(*θ*, *ω*|*ITD*_1:*k*_) over the angular velocity *ω*. Because we are estimating a circular variable, the Bayesian prediction is the direction of the Bayesian prediction vector, defined as the vector that points in the direction of the mean value of direction *n* steps in the future:

$${BV}_{k}=\int \;u\left(\theta \right)p_{k+n}\left(\theta |{ITD}_{1:k}\right)d\theta ,$$

where *u*(*θ*) is a unit vector pointing in direction *θ*.

### Kalman filter prediction

We used the Kalman filter to compute the Bayesian prediction for simulations where the linear approximation to the relationship between direction and ITD was valid. The Kalman filter computes the mean and covariance of the posterior when the system is linear with Gaussian noise [41]. Given that the relationship between azimuth and ITD is nearly linear for the frontal hemisphere, a linear model is a reasonable approximation to our system. The dynamical system for the moving source can be described as:

$$x_{k}=Ax_{k-1}+\varsigma_{k}$$

where the state vector consists of the direction and angular velocity

$x_{k}=\left[\begin{array}{c} \theta_{k}\\ \omega_{k} \end{array}\right]$, the matrix $A=\left[\begin{array}{cc} 1 & \Delta t\\ 0 & 1 \end{array}\right]$ describes the state dynamics, and the noise vector contains the noise for direction and velocity $\varsigma_{k}=\left[\begin{array}{c} \eta_{k}\\ \nu_{k} \end{array}\right]$. The noise at time *k* ≥ 1 is Gaussian with zero mean and covariance matrix *Q* and is uncorrelated across time.

The output of the system is a linear approximation to the mapping from direction to ITD plus noise:

$${ITD}_{k}=Cx_{k}+\xi_{k}$$

where *C* = [2.67 0] and *ξ*_*k*_ is a Gaussian noise process with zero mean and variance *R* that is referred to as the observation error.

The Kalman filter is used to compute the mean and covariance of the posterior at each time. Define ${\hat{x}}_{i|j}$ and Σ_*i*|*j*_ to be the mean and covariance, respectively, of the posterior at time *i* given observations up to time *j*. The mean of the posterior distribution is computed recursively through a process of prediction and updating. The prediction one step ahead in time is computed as

$${\hat{x}}_{k|k-1}=A{\hat{x}}_{k-1|k-1}$$

$$\Sigma_{k|k-1}=A\Sigma_{k-1|k-1}A^{T}+Q.$$

Updating the estimate with a new observation is computed as

$${\hat{x}}_{k|k}={\hat{x}}_{k|k-1}+L_{k}\left[{ITD}_{k}-C{\hat{x}}_{k|k-1}\right]\;\text{and}$$

$$\Sigma_{k|k}=\left(I-L_{k}C\right)\Sigma_{k|k-1}$$

where the Kalman gain is

$$L_{k}=\Sigma_{k|k-1}C^{T}{\left[C\Sigma_{k|k-1}C^{T}+R\right]}^{-1}.$$

When an estimate has been made for the state ${\hat{x}}_{k|k}$, it is possible to use that estimate as a basis for predicting future states at time *k+n*. This requires the estimate at time *k* to be multiplied by the state transition matrix *n* times:

$${\hat{x}}_{k+n|k}=A^{n}{\hat{x}}_{k|k}.$$

The covariance of the posterior at time *k+n* is computed as

$$\Sigma_{k+n|k}=\sum_{m=1}^{n}A^{m-1}Q{\left(A^{m-1}\right)}^{T}+A^{n}\Sigma_{k|k}{\left(A^{n}\right)}^{T}.$$

### Particle filter prediction

We used a particle filter to compute the Bayesian prediction for simulations where the linear approximation to the relationship between direction and ITD was not valid. Particle filtering algorithms are sampling-based approaches to approximating the posterior distribution that are valid for nonlinear and non-Gaussian models [40]. The particle filter algorithm we used was adapted from [49]. The algorithm is given by the following steps:

1. Initialize by selecting *m* particles for direction ${\tilde{\theta }}_{k}^{\left(j\right)}$ and angular velocity ${\tilde{\omega }}_{k}^{\left(j\right)}$ from the prior distribution, *j* = 1, 2, …, *m*. Here we used *m* = 10,000. Set the time index *k* = 1. For each time *k* iterate the following steps.
2. Compute weights for the particles using the likelihood of the ITD measurement at time *k*:
  $$c_{k}^{\left(j\right)}=p\left(ITD_{k}|{\tilde{\theta }}_{k}^{\left(j\right)},{\tilde{\omega }}_{k}^{\left(j\right)}\right)=\;\frac{1}{\sqrt{2\pi \sigma^{2}}}e^{-\frac{1}{2\sigma^{2}}{\left(ITD_{k}-A\;\text{sin}\left(2\pi f{\tilde{\theta }}_{k}^{\left(j\right)}\right)\right)}^{2}}$$
3. Normalize the weights $c_{k}^{\left(j\right)}$ so that the sum is one and draw a new sample of *n* particles with replacement from the previous set of particles, where the probability of selecting particles ${\tilde{\theta }}_{k}^{\left(j\right)},{\tilde{\omega }}_{k}^{\left(j\right)}$ is equal to $c_{k}^{\left(j\right)}$. These new particles represent the posterior distribution at time *k*.
4. The posterior at time *k* + *n* is approximated by propagating the particles according to the source dynamics over *n* steps
  $${\tilde{\theta }}_{k+i+1}^{\left(j\right)}={\tilde{\theta }}_{k+i}^{\left(j\right)}+\Delta t{\tilde{\omega }}_{k+i}^{\left(j\right)}+{\tilde{\eta }}_{j,i}$$
  $${\tilde{\omega }}_{k+i+1}^{\left(j\right)}={\tilde{\omega }}_{k+i}^{\left(j\right)}+{\tilde{\nu }}_{j,i}$$
  for *i* = 0, 1, …, *n*– 1, where ${\tilde{\eta }}_{j,i}$ and ${\tilde{\nu }}_{j,i}$ are linearly independent circular Gaussian and Gaussian noise factors, respectively, as in the generative model.
5. The estimate of the source direction under the predictive posterior *p*_*k*+*n*_(*θ*, *ω*|*ITD*_1:*k*_) is found as the circular mean of the particles ${\tilde{\theta }}_{k+n}^{\left(j\right)}$.
6. Update the time step to *k* + 1 and repeat from step 2 using the particles ${\tilde{\theta }}_{k+1}^{\left(j\right)}$ and ${\tilde{\omega }}_{k+1}^{\left(j\right)}$ found in step 4.

### Neural population model

The neural population model consists of 5000 Poisson neurons with receptive fields that shift according to the prediction given in the proposition proved in the results. The preferred directions of the neurons were drawn from the prior Gaussian distribution with mean zero and standard deviation 23.3 deg. These preferred directions match the model of Fischer and Peña (2011). To generate the neural responses to a sequence of ITD inputs we first computed the predictive posterior *p*_*k*+*n*_(*θ*, *ω*|*ITD*_1:*k*_) as described above. We then used our main result specifying that the activities are proportional to the ratio of the posterior and prior to generate the spiking probabilities for the population of neurons. We scaled the ratio of the posterior to the prior so that firing rates would be approximately 10 spikes/s for neurons with peak responses. Spike counts were generated for the population at each time step using independent Poisson neurons with the specified rate. The direction of the PV was used to estimate the predicted source direction at each time.

The PV was tested for counterclockwise source trajectories with initial directions covering -180 deg to 180 deg in 10 deg steps and angular velocities ranging from 0 deg/s to 150 deg/s in 25 deg/s steps. We calculated the RMS error between the PV estimate *θ*_*PVA*_(*t*) and the Bayesian prediction *θ*_*Bayes*_(*t*) to quantify the approximation error where

$$RMS=\sqrt{\frac{1}{T}\int_{0}^{T}{\left(\theta_{Bayes}\left(t\right)-\theta_{PVA}\left(t\right)\right)}^{2}dt}.$$
